# Supplementary material for: Bias-Reduced Neural Networks for Parameter Estimation in Quantitative MRI
Source: ArXiv. 2024 Feb 26:arXiv:2312.11468v2. Preprint. [Version 2] (PMC10925387)
Supplement: Supplementary file 1 [file NIHPP2312.11468v2-supplement-1.pdf]

Received ; | Revised ; | Accepted
DOI: xxx/xxxx

SUPPORTING INFORMATION

Bias-Reduced Neural Networks for Parameter Estimation in Quantitative MRI

Andrew Mao[1,2,3] | Sebastian Flassbeck[1,2] | Jakob Assländer[1,2]

[1]Center for Biomedical Imaging, Department of Radiology, New York University Grossman School of Medicine, New York, New York
[2]Center for Advanced Imaging Innovation and Research (CAI2R), Department of Radiology, New York University Grossman School of Medicine, New York, New York
[3]Vilcek Institute of Graduate Biomedical Sciences, New York University Grossman School of Medicine, New York, New York

arXiv:2312.11468v2 [physics.med-ph] 26 Feb 2024

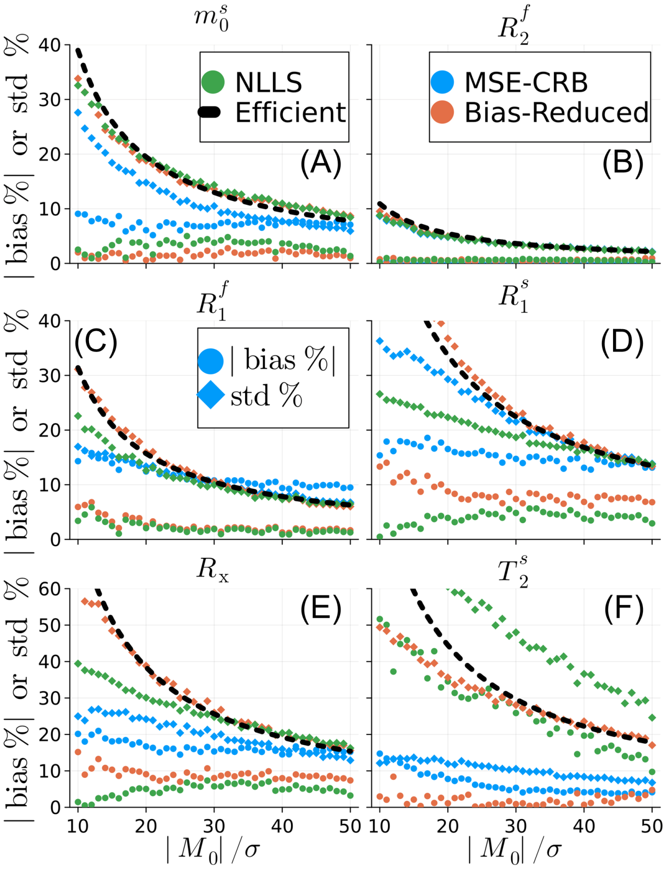

Supporting Figure S1 Repetition of Fig. 2 showing the normalized absolute percent bias and percent standard deviation of the 2-pool qMT parameters in grey matter ($m_0^s = 0.091$, $R_1^f = 0.37/s$, $R_2^f = 11.9/s$, $R_x = 20.5/s$, $R_1^s = 3.17/s$, $T_2^s = 11.7\mu s$) [17] as a function of SNR ($|M_0|/\sigma$) for neural networks trained using the MSE-CRB [15] and proposed Bias-Reduced losses in comparison to non-linear least squares (NLLS) and a hypothetical efficient estimator. Here, similarly to white matter, the proposed strategy performs similarly to NLLS in all parameters except T_2^s, where the performance is more in line with an efficient estimator.

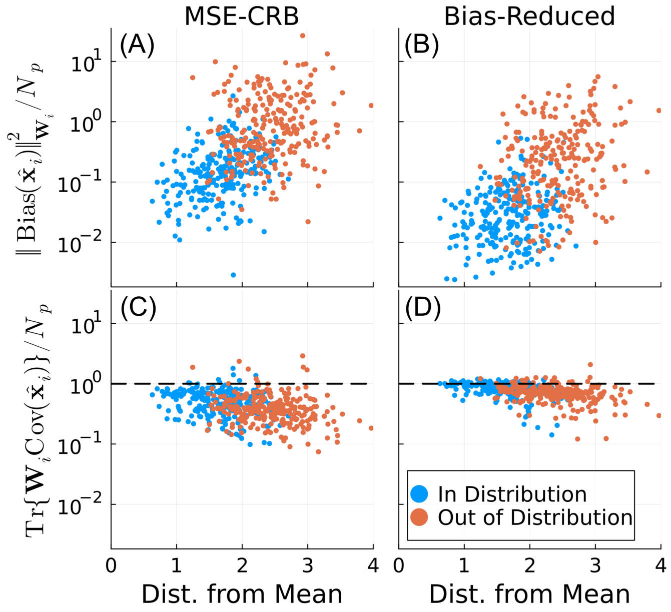

Supporting Figure S2 Comparison of the MSE-CRB and Bias-Reduced NN's CRB-weighted squared bias and variance for 500 qMT fingerprints (each with a random SNR) randomly sampled from a mixture of Gaussian distributions truncated at non-physical values (e.g., constraints like $0 \leq m_0^s \leq 1$ are still imposed). Fingerprints that are outside the cutoff ranges of the training data distribution in any parameter are colored red, and otherwise blue. The x-axis shows the Euclidean distance from the mean of the training distribution, weighted by the standard deviations (e.g., calculated from z-scores of the individual parameters), and thus (approximately) follows the Chi distribution. The dashed black line corresponds to the Cramér-Rao Bound. For both networks, the bias is generally higher for fingerprints outside the training distribution. The proposed training strategy reduces the overall bias for fingerprints both in and outside of the distribution.

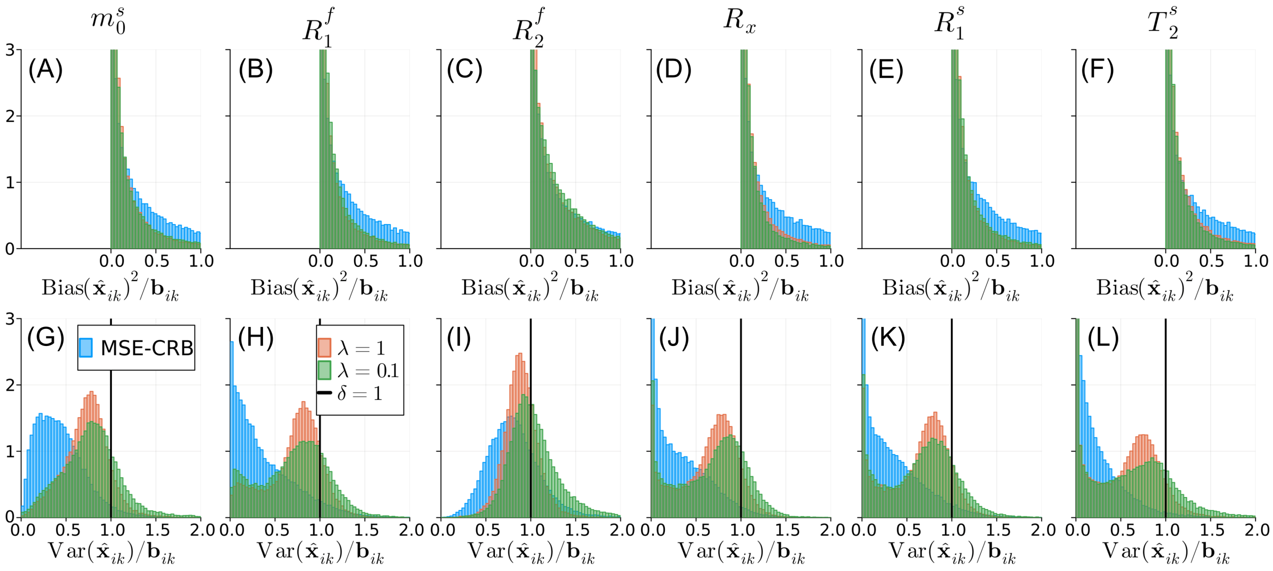

Supporting Figure S3 Repetition of Fig. 3 where all fingerprints in the test set have a random SNR higher than the range of SNRs seen during training. In this case, the bias is overall higher for both networks. While this suggests somewhat impaired generalization of the employed NN architecture,[50] it is also consistent with normalization by smaller Cramér-Rao bounds—which account for the decreased noise level—and an expected floor to the accuracy of the NN estimator that is related purely to measurement noise. The proposed strategy for reducing bias still holds outside of the training range of SNRs, albeit somewhat less for R_2^f.

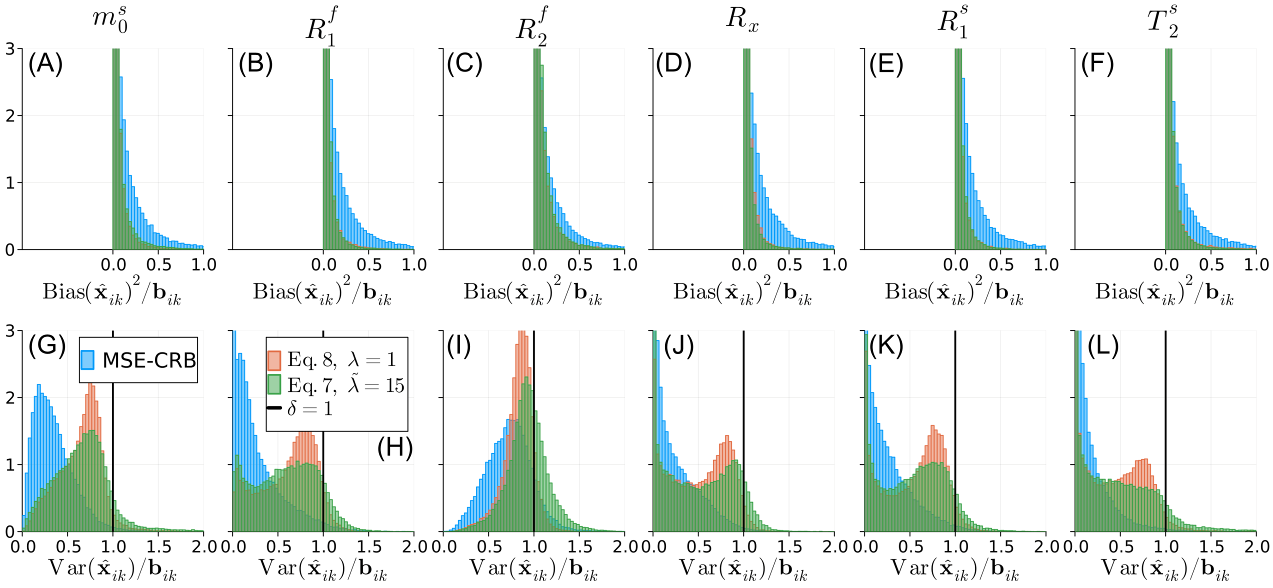

Supporting Figure S4 Repetition of Fig. 3 comparing NNs trained with the MSE-CRB, the proposed Bias-Reduced and the bias-constrained loss (Eq. (7)) with an optimized lambda. While the bias-constrained approach has similar bias to the Bias-Reduced strategy, it has less uniform variance properties across all estimated qMT parameters with a longer tail past the $\delta = 1$ line.

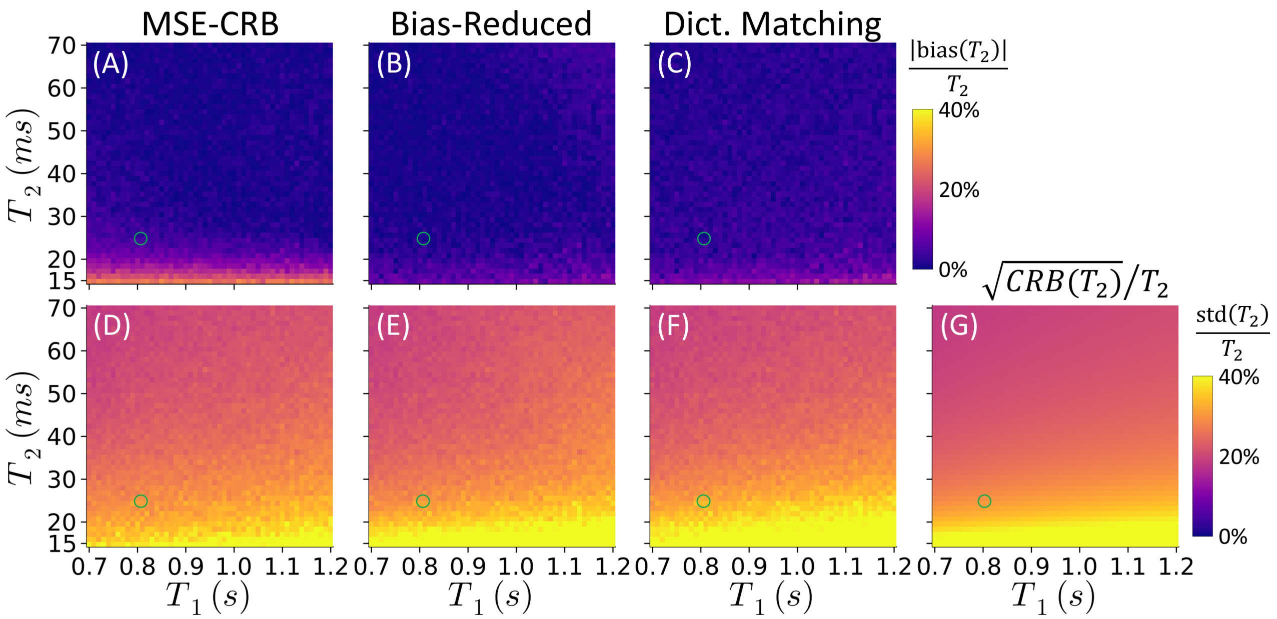

Supporting Figure S5 Normalized bias and standard deviation of the FISP-based T_2 estimates as a function of T_1 and T_2 using NNs trained with two different strategies in comparison to a dictionary-matching-based reference (C,F). (A,D) The Cramér-Rao bound weighted mean squared error (MSE-CRB).[15] (B,E) The Bias-Reduced strategy achieves the lowest overall bias throughout parameter space with a similar variance to the CRB reference (G). The green circle marks the average white matter values measured in vivo (Fig. 5).

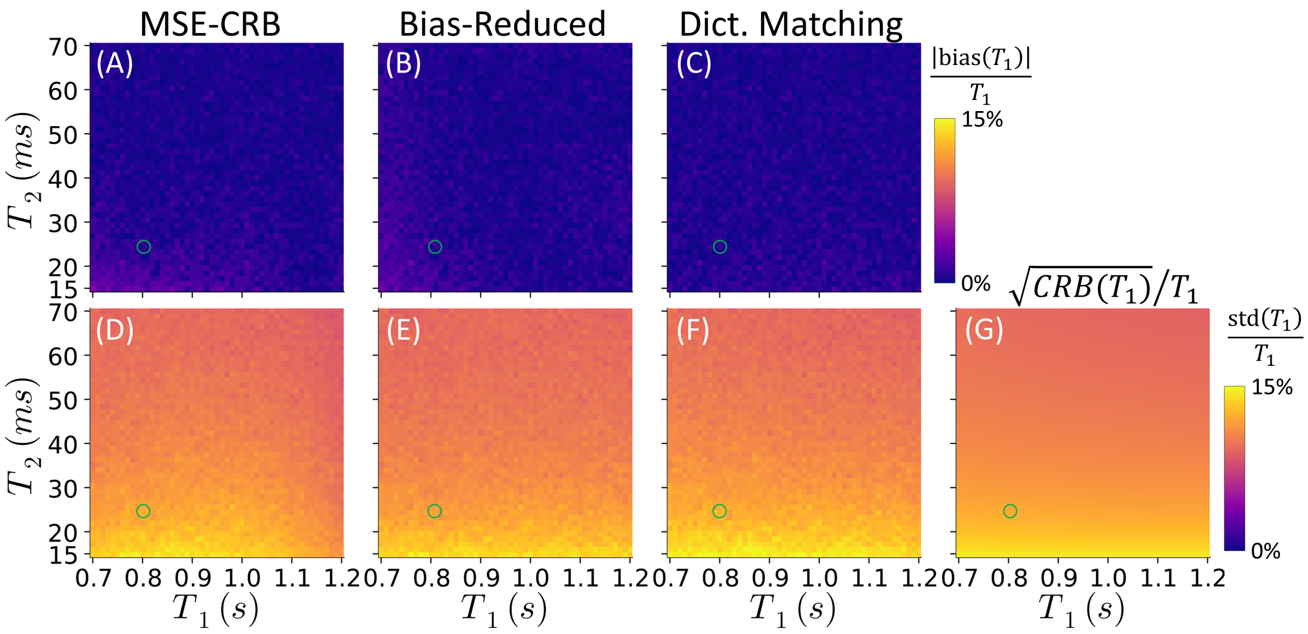

Supporting Figure S6 Repetition of Sup. Fig. S5 showing the normalized absolute percent bias and percent standard deviation of the FISP-based T_1 estimates instead. In this case, the performance is similar between NNs trained using both strategies and the reference.

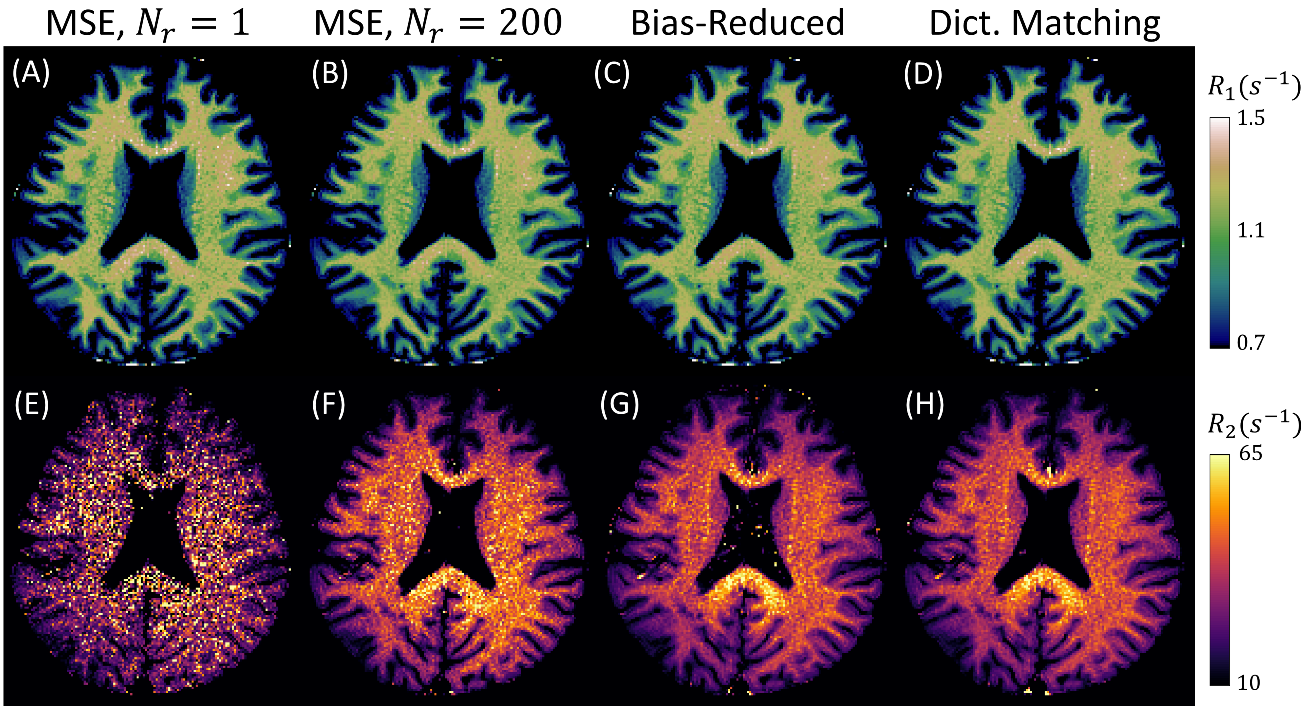

Supporting Figure S7 Comparison of in vivo FISP $1/T_1$ and $1/T_2$ maps estimated using NNs trained with the typical mean squared error (MSE) criterion in comparison to the proposed method and dictionary matching. With only one noise realization ($N_r = 1$), small T_2 values are poorly represented in the overall MSE loss, contributing to poor T_2 fits in vivo (E, consistent with Fig. 5 of Ref. 8). While this is somewhat mitigated by averaging over $N_r = 200$, the resulting T_2 maps are still biased (F), which is ameliorated by use of the proposed Bias-Reduced strategy (G).
